# Genotypic drug resistance and transmission clusters of *Mycobacterium tuberculosis* isolates among Ethiopian returnees from Saudi Arabia

**DOI:** 10.1371/journal.pone.0318743

**Published:** 2025-04-16

**Authors:** Melaku Tilahun, Abay Atnafu, Tewodros Tariku Gebresilase, Muluye Abebe, Mekdes Alemu, Sebisib Neway, Taye Letta, Abiot Bezabeh, Tamiru Assefa, Kalkidan Melaku, Dawit Hailu Alemayehu, Shewki Moga, Abaysew Ayele, Maeruf Fetu, Bethlehem Adnew, Andargachew Mulu, Liya Wassie, Kidist Bobosha

**Affiliations:** 1 Armauer Hansen Research Institute (AHRI), Addis Ababa, Ethiopia; 2 Ministry of Health (MOH), Addis Ababa, Ethiopia; 3 REACH Ethiopia, USAID-Urban TB LON Project. Addis Ababa, Ethiopia; 4 Ethiopian Public Health Institute (EPHI), Addis Ababa, Ethiopia; The University of Georgia, UNITED STATES OF AMERICA

## Abstract

**Background:**

Human migration significantly contributes to the global spread of infectious diseases. In recent years, Ethiopia has experienced an increased influx of returnees from the Kingdom of Saudi Arabia. These migrants were often held in densely populated and inadequately ventilated detention centers and faced a heightened risk of tuberculosis transmission. This study investigates the genotypic drug resistance and transmission clusters of *M. tuberculosis* among returnees from various detention centers in the Kingdom of Saudi Arabia. Whole genome sequencing (WGS) is employed to characterize the genotypic drug resistance patterns and transmission clusters of *M. tuberculosis* isolates in presumptive TB cases.

**Methodology:**

Following symptom screening for presumptive TB, sputum samples were collected from 161 Xpert MTB/RIF-confirmed returnees between August and December 2022. The samples were further cultured on Lowenstein-Jensen media, with 66.5% (107/161) yielding positive results. The *M. tuberculosis* strains were classified, and genotypic drug susceptibility was predicted. Transmission clusters were identified using a distance threshold of 12 single nucleotide polymorphisms (SNPs).

**Results:**

Among the 88 isolates, Lineage 4 was the most prevalent, representing 65.9% of *M. tuberculosis* cases among returnees. Sub-lineage 4.2.2.2 was the most dominant within this group, comprising 33% (29/88) of the isolates. Among the isolates, 40 gene mutations conferring resistance to first- and second-line anti-TB drugs were identified. Sixteen transmission clusters were identified, suggesting possible transmission events and a likely origin within the detention centers of the Kingdom of Saudi Arabia (KSA). The proportion of MDR-TB among newly diagnosed cases was 2.3% (2/88).

**Conclusion:**

The clustering patterns of *M. tuberculosis* strain among KSA returnees possibly suggest increased transmission rates in congregate settings. Most importantly, the identified prevalence of MDR-TB, particularly among newly diagnosed TB cases, underscores the need to strengthen robust screening practices for returning migrants before they reintegrate into the community to curb TB/DR-TB transmission.

## Introduction

Tuberculosis (TB) caused by the *Mycobacterium tuberculosis* (*M. tuberculosis*) complex remains a major global public health problem. Approximately 25% of the global population carries *M. tuberculosis*, with a 5–10% chance of developing TB disease over their lifetime [1]. Migration significantly influences the transmission dynamics of TB. Studies highlight migrants’ heightened risk of TB due to factors like limited healthcare access, poor nutrition, and unsafe living conditions in host countries [2]. In addition, the emergence of resistance to anti-TB drugs, particularly multi-drug resistant TB (MDR-TB) has been identified as one of the major problems in migrant populations [3]. Efficient strategies for identifying TB and drug-resistant TB (DR-TB) cases, employing highly sensitive screening tools and rapid diagnostic tests, are crucial in key and at-risk populations such as immigrants. These measures help close the gap between TB incidence and case notification [4].

Saudi Arabia, a dynamic immigration hub with 8.4 million expatriates, presents a unique environment for potential TB transmission among immigrants [5]. Over the past two decades, Ethiopian migration to the Middle East, especially Saudi Arabia, has surged. Current Ethiopian Ministry of Foreign Affairs data shows over 750,000 Ethiopians live in Saudi Arabia, with over half lacking proper documentation [6]. Ethiopian migrant workers prefer these destinations for job opportunities, with many undertaking illegal journeys to KSA driven primarily by economic motivations [7]. With the onset of the COVID-19 pandemic, KSA took active measures to detain undocumented migrants and placed Ethiopian migrants in overcrowded detention centers, lacking proper ventilation. These conditions increase the risk of infectious diseases, including TB [8], subsequently resulting in mass deportation, which further poses a significant public health challenge, as these immigrants are often labeled as key and at-risk populations for TB [9].

This mass deportation presented a significant public health challenge, notably due to the risk of introducing a high number of TB cases, potentially including MDR-TB strains, to the Country. This influx could exert significant pressure on Ethiopia’s healthcare system, particularly amidst the ongoing conflict. Therefore, as part of infection prevention strategies and to control TB, the Ministry of Health of Ethiopia set up a task force that is responsible for screening and conducting clinical and bacteriological tests on these migrants before they assimilate into their communities. This study aims to characterize *M. tuberculosis* isolates from presumptive TB cases among returning migrants using a whole genome sequence (WGS) approach, with a focus on molecular epidemiology, drug resistance, and transmission clusters.

## Methodology

### Study setting

This was a cross-sectional study, conducted among Ethiopian migrants returned from KSA to Ethiopia between August and December 2022. The study focused on individuals suspected of having pulmonary tuberculosis (PTB) among the returnees who stayed in different detention centers across the KSA. Returnees who had spent extended periods in detention centers in KSA were scheduled to return to Ethiopia as part of an agreement between the Federal Democratic Republic of Ethiopia and KSA. Returnees presenting with TB symptoms and/or chest X-ray abnormalities suggestive of TB were asked to provide sputum samples for bacteriological diagnosis using the Xpert MTB/RIF assay. For those confirmed with TB by the assay, additional sputum specimens were collected and sent to Armauer Hansen Research Institute (AHRI) for culture isolation of the *M. tuberculosis* complex (MTBC) and Whole Genome Sequencing (WGS). All diagnosed individuals were linked to the Chefe Health Center for initiation of directly observed treatment short course (DOTS) and were isolated at the center for 15 days, the recommended period after which they are considered non-infectious. Subsequently, returnees were transferred to health facilities in their residential areas to complete the standard treatment duration.

### *M. tuberculosis* culture and identification

Sputum samples were processed for *M. tuberculosis* culture isolation at AHRI using the NALC-NaOH method, following established protocols. The sediment was resuspended in 1.5 ml phosphate-buffered saline and inoculated into conventional LJ media slants, supplemented with 0.4% sodium pyruvate and 0.3% glycerol to support growth. The inoculated slants were incubated at 37°C for a minimum of 8 weeks, with weekly observations for mycobacterial colonies. Ziehl-Neelsen staining was used to microscopically examine colonies for Acid-Fast Bacilli (AFB) positivity [10]. A portion of the AFB-positive colonies was heat-killed at 80°C for 20 minutes for mycobacterial DNA extraction, while the remaining colonies were frozen in duplicate glycerol stocks at −80°C. These replicate bacterial stocks were stored separately at the central AHRI TB laboratory as a backup. Heat-inactivated isolates were examined using Polymerase Chain Reaction (PCR) based deletion typing to identify the presence of a region of deletion (RD) 9, differentiating *M. tuberculosis* complex (MTBC) from other mycobacterial species [11].

### Genomic DNA extraction and purification

Bacterial DNA was extracted and purified using the cetyltrimethylammonium bromide (CTAB) method. Briefly, 400 µl of heat-killed suspension was added to an Eppendorf tube with 50 µl of 10 mg/ml lysozyme and incubated at 37°C for 1 hour. Next, 75 µl of 10% SDS was mixed in, followed by 50 µl of proteinase K, and incubated at 65°C for 10 minutes. Then, 100 µl of 5M NaCl and prewarmed CTAB/NaCl solution were added until the solution turned milky, and incubated at 65°C for 10 minutes. After adding 750 µl of chloroform: Isoamyl alcohol (24:1) and centrifuging at 11,000×g for 8 minutes, the top aqueous phase was collected, and DNA was precipitated with alcohol [12]. Finally, the DNA pellet was dissolved in molecular-grade water. DNA quality was assessed by running the sample on a 1% agarose gel and quantified using a Qubit fluorometer.

### Library preparation and whole genome sequencing

Genomic DNA was used as input for the library preparation. Small DNA fragments and dimer primers were removed during the library prep using Ampure XP beads (BECKMAN COULTER Life Sciences, USA, Cat. No. A63881) after adapter ligation and indexing PCR, respectively, according to the manufacturer’s library preparation kit (NEW ENGLAND Biolabs, USA, Cat. No. E7103). Briefly, 100–500 ng of the genomic DNA was fragmented mechanically using Covaris Ultra Sonicator (Covaris, M220) and ligated with DNA adapter sequences. The reaction product was cleaned by Ampure XP beads following the manufacturer’s protocol. Five-cycle-PCR was used to add index 1 (i7) and index 2 (i5) to uniquely identify each sample. Following amplification, the libraries were cleaned, and the concentration was measured using a Qubit 4.0 fluorometer (Life Technology Holdings, Singapore). Selected libraries from each batch of library preparations were analyzed based on the fragment size using the Bioanalyzer 2100 fragment analyzer (Agilent, Germany). Finally, up to100 libraries that passed the concentration and fragment size quality control were pooled and sequenced on NextSeq550 (Illumina, Singapore) high output kit (Illumina, USA, Cat. no. 20024908) for a PE 150 cycle sequencing [13] at the Ethiopian Public Health Institute (EPHI) sequencing facility.

### Bioinformatics analysis

The quality of paired-end reads was assessed and subsequently trimmed with Trimmomatic (version 0.39) to remove adapters, low-quality bases (phred score <20), and short reads of less than 40 base pairs [14]. Contamination was checked with kraken2 and reads with less than 10% contamination were considered for further analysis [15]. Reads were aligned to the *M. tuberculosis* H37Rv strain reference genome (NC_000962.3). Samtools was used to sort and index aligned files and a minimum average depth of 20 was taken as threshold [16]. Variants were called using bcftools, and SNPs in the repetitive PE/PPE regions were masked. Drug resistance and lineage profiles were predicted using the TB-Profiler (version 6.2.0) database [17]. A SNP-based phylogenetic tree was then constructed using IQ-TREE maximum likelihood models, with a bootstrap value of 1000 replications [18]. Tree visualization and annotation were performed using the iTOL (Interactive Tree Of Life) web servers [19]. The cluster graph was reconstructed and visualized using the GraphSNP transmission analysis tool [20]. Pairwise SNP distances were calculated with the snp-dists Python package, applying a cutoff threshold of 12 SNPs [21]. A breadth-first search (BFS) algorithm identified cluster memberships, while Kruskal’s algorithm constructed the minimum spanning tree (MST) of the clusters[22,23]. Raw sequence data was submitted to the Sequence Read Archive and can be found under NCBI BioProject ID PRJNA1155881.

### Epidemiological and genomic cluster analysis

Health professionals appointed by the Federal Ministry of Health (FMoH) of Ethiopia conducted a thorough epidemiological investigation on the returnees’ age, sex, prior addresses, and time spent at the detention centers. The primary goal was to identify epidemiological linkages among returnees using a standardized TB screening tool. The procedure prioritized direct collection of vital epidemiological details directly from returnees, including detention center locations, living conditions, and duration of stay. This approach streamlined the identification of possible transmissions, supported by sequence analysis. Genomic clusters were identified independently of epidemiological data, defined by instances where the genetic distance between isolates from returnees, measured by SNPs, did not exceed 12 [21,24].

### Data management and analysis procedure

Sociodemographic and clinical data were recorded using a paper-based standardized TB screening form, which was later transported to AHRI. Two independent data encoders entered the data into Excel using the double-entry method to ensure accuracy. The data was then checked for consistency. Before exporting to a Comma-Separated Values (CSV) file, all personally identifiable information was removed to prevent author access to such data during the research. The anonymized CSV file was imported into R (version 4.3.2) for cleaning and statistical analysis. Descriptive statistics were used to calculate variable frequencies and percentages.

### Ethical approval

Ethical approval was obtained from the AHRI/ALERT Ethics Committee (Protocol No. PO26/20 22). All participants provided informed consent before their inclusion into the study. Additionally, a support letter was sought from the Ministry of Health. Before enrollment, participants received a standardized information sheet outlining the study’s objective, risks, and benefits. Questions were addressed, and those who consented signed an informed consent form before enrollment. Based on the result all individuals who were identified as TB and RR-TB positive were treated according to the National TB treatment guidelines.

## Results

### Sociodemographic characteristics and clinical presentations

The median age of the study participants was 25 years (IQR = 22–29) and all were males, who initially migrated to KSA from Tigray, Oromia, Amhara, and Afar regional states in Ethiopia. Over half of the returnees reported cardinal TB symptoms, including persistent cough, low-grade fever, night sweats, and loss of appetite (S1 Table). The average duration of stay for these returnees was 36.6 months (IQR = 24–48 months) (S1 File).

### Mutations driving first- and second-line anti-tb drug resistance

**Fig 1:**
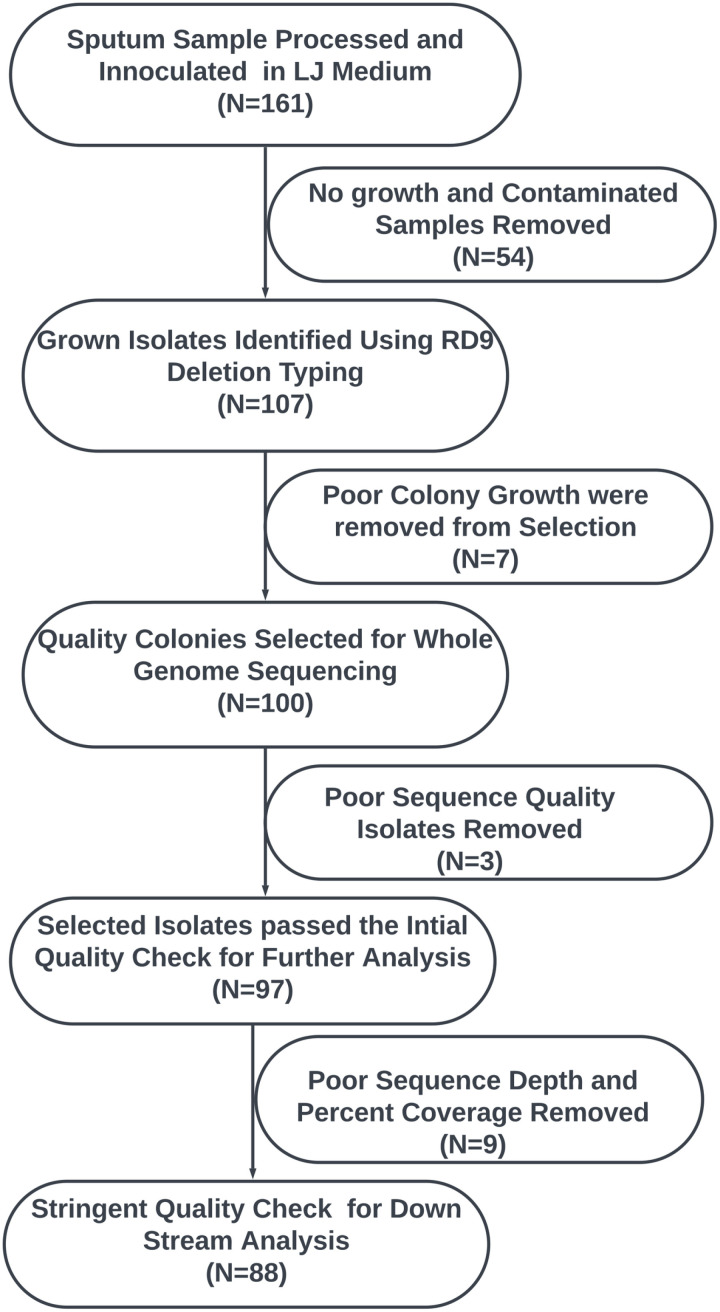
Sample selection and quality control for *M. tuberculosis* WGS.

*M.tuberculosis* isolates from 161 Xpert MTB/RIF-confirmed PTB participants underwent LJ culture and further species identification. Among these, 107 (66.5%) tested positive for *M. tuberculosis*, and 100 were selected for sequencing based on DNA quality control (S1 File). Of these, 88 isolates passed the quality checks for downstream analysis using WGS (S1 File) (Fig 1). Among the isolates studied, we identified 40 gene mutations conferring resistance to both first- and second-line anti-TB drugs (Table 1). Mutations linked to isoniazid (INH) resistance comprised 25% (10/40) of the drug resistance mutations identified. The majority of these (90%, 9/10) exhibited the Ser315Thr mutation in the *katG* gene. A distinctive mutation, Ala424Val, was identified within the *katG* gene in one isolate, marking a divergence from the commonly observed INH resistant mutations. No INH resistance-associated mutations were identified in the *inhA* gene. Streptomycin (STR) resistance was identified in 18.2% (16/88) of the isolates, with mutations present in the *gid*, *rpsL,* and *rrs* genes. Ethambutol resistance was identified in 2.3% (2/88) of the isolates, associated with the Met306Ile substitution in the *embB* gene. A mutation in the *fbiC* gene, specifically c.2547–2565dup, was detected in a single isolate conferring resistance to the newly incorporated second-line anti-TB drugs, Delamanid (DLM) and Pretomanid (PMD). Resistance to Pyrazinamide (PZA), Capreomycin (CAP), and Ethionamide (ETH) were identified with corresponding mutations found in the *pncA*, *tlyA*, and *ethA* genes, respectively. Rifampicin (RIF) resistance, driven by the Ser450Leu mutation in the *rpoB* gene, was identified, contributing to the observed 3.4% prevalence of MDR-TB among the isolates. Within this subset of MDR-TB, 2.3% (2/88) were detected in newly diagnosed individuals, while 1.1% (1/88) had prior TB treatment history.

**Table 1:** *M. tuberculosis* gene mutations linked to anti-TB drug resistance.

Drugs	Genotypic drug resistance prediction				Total N (%)
	Target Gene	Mutation	Non-MDR-TB N (%)	MDR-TB N (%)	
**Rifampicin**	***rpoB***	Ser450Leu		3 (100)	3 (3.4)
**Isoniazid**	***katG***	Ala424Val	1 (10)		10 (11.4)
		Ser315Thr	6 (60)	3 (30)	
**Pyrazinamide**	***pncA***	c.-11A>G		2 (100)	2 (2.3)
**Ethambutol**	***embB***	Met306Ile		2(100)	2 (2.3)
**Streptomycin**	***gid***	c.102delG	2 (28.6)		16 (18.2)
		c.87delC	3 (42.8)		
		p. Gly69Asp		2 (28.6)	
	***rpsL***	p. Lys43Arg		1(100)	
	***rrs***	n.888G>A	5(71)	2(29)	
		n.799C>T	1(100)		
**Capreomycin**	***tlyA***	p. Asn236Lys	2 (100)		2 (2.3)
**Ethionamide**	***ethA***	c.1341delC		1 (100)	3 (3.4)
		p. Met1?		2(100)	
**Delamanid**	***fbiC***	c.2547–2565dup		1(100)	1 (1.1)
**Pretomanid**	***fbiC***	c.2547–2565dup		1(100)	1 (1.1)

### *M. tuberculosis* lineages and drug resistance patterns

**Fig 2:**
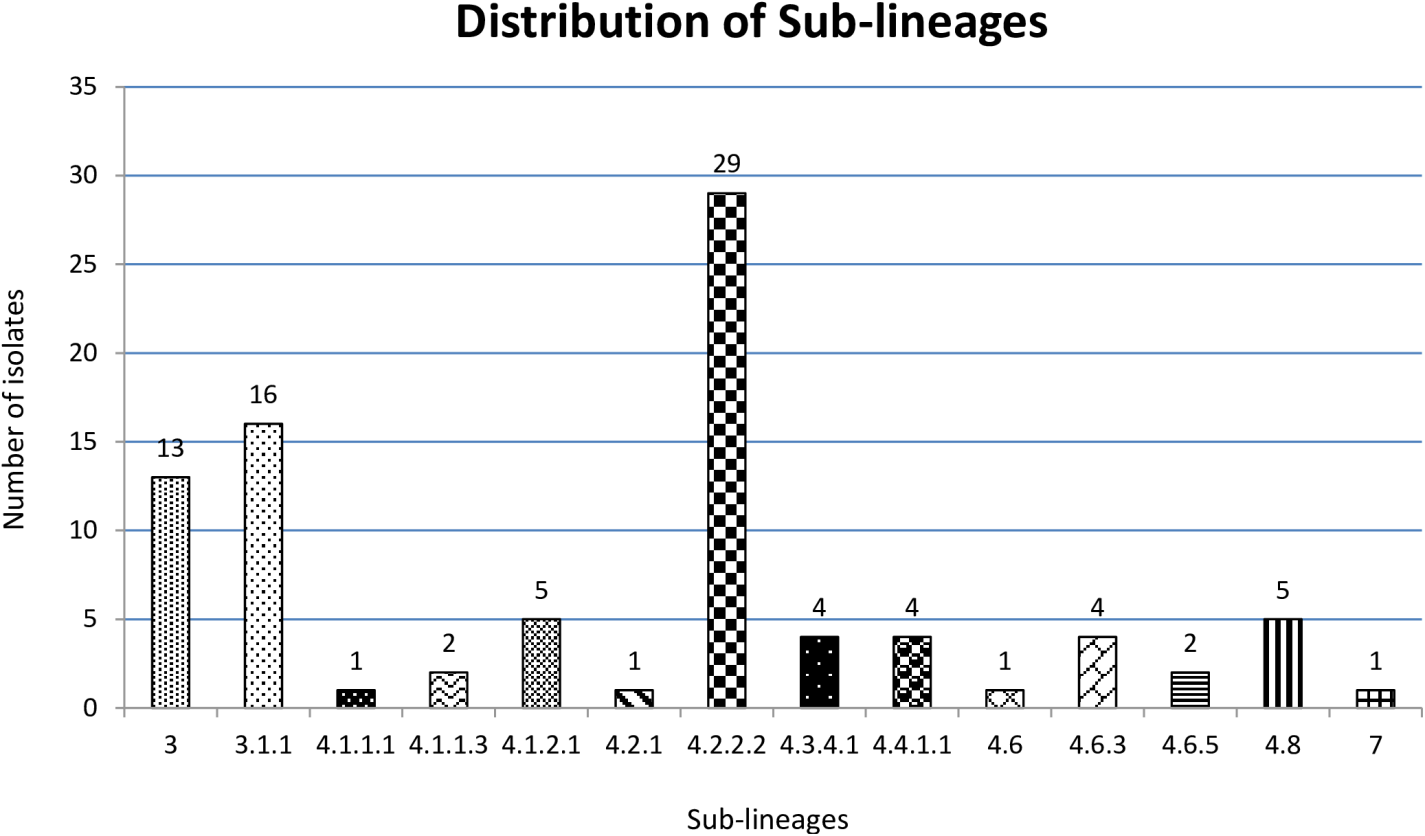
Sub-lineage distribution of *M. tuberculosis* among the returnee.

Our analysis identified 14 distinct sub-lineages. The most prevalent was sub-lineage 4.2.2.2, accounting for 33% (29/88) of the cases. This was followed by sub-lineage 3.1.1 at 18.2% (16/88) and sub-lineage 3 at 14.8% (13/88) (Fig 2).

Compared to other lineages, lineage 4 exhibited a markedly higher prevalence of drug resistance, where 27.6% (16/58) of the lineages were resistant to at least one anti-TB drug (Table 2).

**Table 2:** Anti-TB drug resistance patterns among *M. tuberculosis* lineages.

Drug resistance pattern	Number of isolates per lineage		
	Lineage 3	Lineage 4	Lineage 7
**CAP**	–	1	–
**Hr-TB**	–	2	–
**INH+CAP**	–	1	–
**INH+DLM**+**PMD**	–	1	–
**INH+STM**	–	3	–
**MDR**	–	3	–
**STM**	2	5	–
**Susceptible**	27	42	1
**Total**	29	58	1

CAP = Capreomycin, Hr-TB = Isoniazid-resistant, INH = Isoniazid, DLM = Delamanid, PMD = Pretomanid, STM = Streptomycin, MDR = multi-drug resistant

### Phylogenetic analysis and identification of transmission clusters

**Fig 3:**
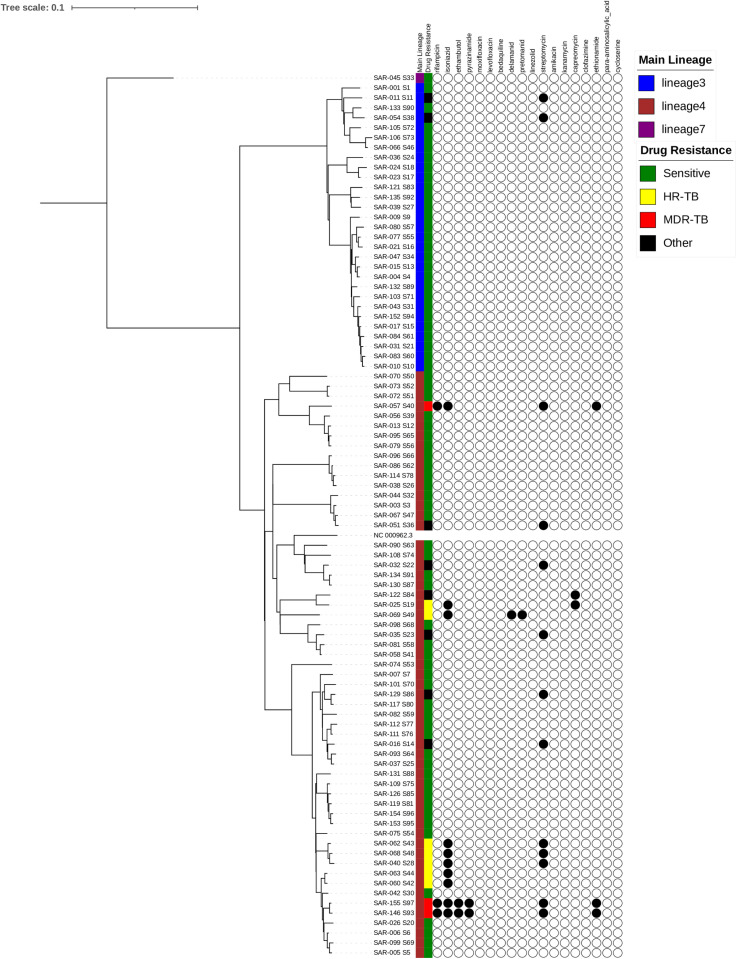
A maximum likelihood phylogenetic tree was created using IQ-TREE, with a bootstrap support value exceeding 85%. This tree, based on 88 M. tuberculosis isolates collected from returnees between August and December 2022, illustrating their lineages and genotypic resistance to both first- and second-line anti-TB drugs. The tree was annotated using iTOL v6 (https://itol.embl.de/). The first column denotes the main lineages, while the second column shows the drug resistance predictions for the isolates. Resistance-associated mutations are depicted by filled circles (presence of mutation against specific drug) or empty circles (absence of mutation). MDR refers to multi-drug-resistant, Hr-TB to isoniazid-resistant, and ‘other’ includes mono- and poly-resistant strains.

Phylogenetic analysis of *M. tuberculosis* identified lineages 3 and 4 as the most circulating *M.tuberculosis* strains, with a significant proportion belonging to lineage 4. Additionally, the Ethiopia-specific lineage, lineage 7 was also found among the returnees (Fig 3).

**Fig 4:**
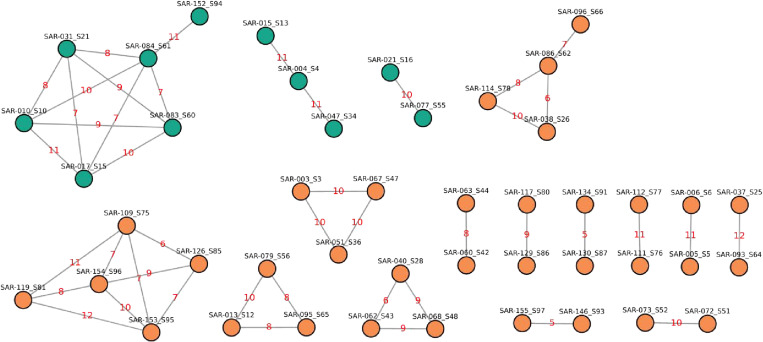
Transmission network and clusters of *M. tuberculosis* lineages among returnees. Green nodes represent lineage 3 clusters, while orange nodes represent lineage 4 clusters. The red-highlighted numbers represent the genetic distances between isolates, calculated based on Single Nucleotide Polymorphisms (SNPs).

Out of the 88 sequenced *M. tuberculosis* isolates, a significant proportion, 51.1% (45/88), showed 16 putative transmission clusters (S1 File). These clusters, characterized by paired sizes ranging from two to six individuals, were distinguished through the application of a single nucleotide polymorphism (SNP) distance cut-off ≤ 12 (Fig 4) (S1 File). Significantly, clusters emerging from diverse detention centers across KSA revealed an epidemiological link, indicating shared locations during participants’ detention periods (S1 File). One of the clusters, which includes samples SAR-109_S75, SAR-119_S81, SAR-126_S85, SAR-153_S95, and SAR-154_S96, formed with a SNP distance ranging from 6 to 12. These samples were collected from returnees who were detained within the same facility during their stay in KSA.

**Fig 5:**
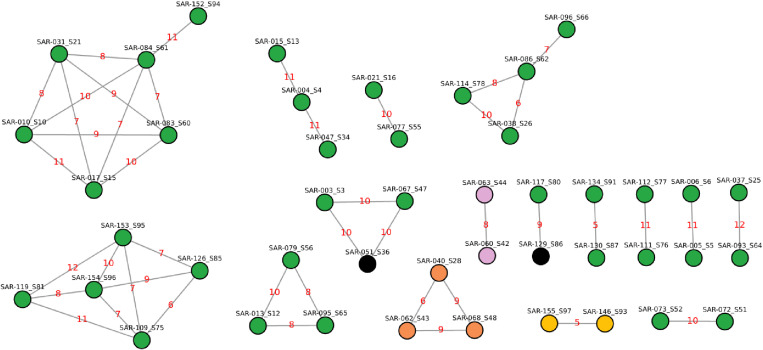
Transmission network and clusters of drug-resistant *M. tuberculosis* isolates among returnees. Green nodes represent susceptible isolates, purple nodes indicate Hr-TB isolates, black nodes correspond to STM-resistant isolates, yellow nodes represent MDR isolates, and orange nodes indicate INH+STM-resistant isolates. The red-highlighted numbers represent the genetic distances between isolates, calculated based on Single Nucleotide Polymorphisms (SNPs).

Clusters with drug-resistant paired isolates were also identified with a SNP distance of less than 12, suggesting recent transmissions of drug-resistant isolates (Fig 5). The MDR-TB strain identified in samples SAR-146_S93 and SAR-155_S97 was collected from individuals residing in the same detention center during their stay in KSA (S1 File). Notably, SAR-155_S97 has a reported history of prior anti-TB treatment, while SAR-146_S93 was new to any anti-TB treatment (S1 File), further highlighting the significance of detention centers as potential hubs for recent TB and MDR-TB transmission.

## Discussion

A WGS approach was used to explore the molecular epidemiology, and transmission clusters of *M. tuberculosis* among Ethiopian returnees from KSA, who had been staying in crowded detention centers, a condition that often facilitates TB transmission. Our research revealed the presence of drug-resistant mutations in a significant proportion of *M. tuberculosis* isolates, particularly those resistant to isoniazid. In this study we noted that MDR-TB cases among the study participants did not receive prior anti-TB treatment, suggesting potential transmission and acquisition of drug-resistant strains within the study setting. These findings underscore the need for targeted interventions and policies aimed at preventing the dissemination of TB/DR-TB strains to ensure successful community assimilation of these returnees.

Notably, despite female participants undergoing Xpert MTB/RIF testing, our study exclusively consisted of male participants. Male migrant workers commonly work in skilled and professional services, while female migrants typically serve in private homes, undertaking roles such as domestic caregivers, housekeepers, cleaners, and cooks [25]. In recent years, KSA intensified the detention and deportation of undocumented workers, and males were disproportionately affected. Recent International Organization for Migration (IOM) data showed a 34% increase in male returnees from KSA since 2021, contrasted with a 29% decrease in female returnees [26]. The information provided by the detainees indicated that the detention conditions were overcrowded and poorly ventilated, which significantly contributes to the facilitation of TB transmission. This context likely explains the observed increase in TB infection prevalence among male participants in this study. However, while this could be the case, the absence of female participants from this study may not reflect the reality in the actual burden of the disease among females.

This study also reveals a rich spectrum of mutations associated with drug resistance, prominently observed within the *katG* gene, a key determinant of INH resistance [27]. Among the 40 mutations identified in drug resistance-conferring genes across all isolates, the second largest number was linked to INH, a key first-line anti-TB treatment [28]. Notably, the *katG* gene exhibited 10 occurrences of mutations. Remarkably, in this study, a previously unreported mutation, the Ala424Val substitution in the *katG* gene, which is absent in the 2023 Catalogue of Mutations in *M. tuberculosis* Complex and their association with drug resistance guidelines [29], was identified. This observation possibly highlights the emergence of novel drug resistance in Ethiopia, emphasizing the critical importance of continuous genomic surveillance. Such surveillance is also vital for identifying and assessing the impact of mutations on TB treatment efficacy, virulence, and transmission clusters within the population [30], ultimately guiding effective control strategies.

A major finding of this study is the identification of mutations conferring resistance to streptomycin (STR) in 18.2% of the isolates. The presence of these mutations, despite this drug being discontinued in Ethiopia since 2005 as a first-line treatment, suggests there is ongoing transmission of these resistant strains or de novo mutations in the absence of drug pressure [31]. These findings emphasize the need for continued surveillance and assessment of the efficacy of current treatment regimens against such strains. Despite Ethiopia’s recent transition out of the high MDR-TB burden countries [32], the detected 3.4% prevalence of MDR-TB, including a 2.3% rate among new cases, among the returnees sends an alarming signal. These figures are notably higher than the national average and exceed those reported in similar studies [33]. Overall, the identification of new and existing mutations, alongside the persistence of resistance to discontinued drugs, underscores the evolving TB challenge and calls for intensified public health surveillance and measures to tackle DR-TB, particularly in populations at high risk, such as these returnees.

In recent years, studies have delved into the exploration of DR-TB within refugee settings. These environments bear resemblances to the overcrowded and poorly ventilated conditions often found in detention centers, which are the primary places of origin for the study participants. Recently, *Abyot et al.*, reported a higher prevalence of lineage 3 strains, particularly among South Sudanese and Sudanese refugees [34] whereas our study indicates a predominance of lineage 4 strains. These lineages are frequently reported from different regions of Ethiopia, each exhibiting varying proportions [35,36]. The detection of lineage 7 in returnees likely indicates reactivation rather than new infection. This *M. tuberculosis* lineage is confined to Ethiopia, originally identified in the northern part of Ethiopia, and there have been no reports of this lineage in KSA since 2021[37,38]. Overall, the variation in lineage and strain, despite comparable high-risk settings, possibly suggests the influence of genetic diversity, migration patterns, and possible prior TB exposure on prevalence differences.

The findings also revealed a notable proportion of heightened drug resistance in lineage 4, consistent with the recognized characteristics of contemporary lineages [39]. This aligns with recent reports from northwest Ethiopia, where similar lineages have demonstrated an increased propensity for drug resistance [40]. This raises concerns about the potential for ongoing TB transmission in overcrowded settings, like detention centers and refugee camps, which can be hotspots for the spread of TB, particularly drug-resistant strains. While this study did not identify novel drug-resistant strains, the discovery of the L9 strain among Somali refugees near the Ethiopia-Somalia border, a strain previously confined to Somalia, signals the alarming potential for the cross-border spread of novel TB strains [34]. This highlights the importance of genomic surveillance in tracking the dynamics of TB transmission and resistance patterns in such high-risk areas and underscores the need for further research to inform effective TB control measures in these contexts.

This study investigated isolate clustering, revealing patterns that possibly suggest recent and active transmission of TB within the community [41]. Clustering possibly suggests ongoing transmission, while unique patterns suggest reactivation or recent introduction of *M. tuberculosis* strains into the geographic area [35]. In this study, we found clustering, which may suggest TB transmission, supported by both epidemiological links and genomic analysis revealing SNP distances less than 12. This pattern aligns with findings from similar studies among refugees in shared refugee camps [34] and individuals residing in close localities [42]. On the other hand, while congregated settings, such as detention centers may contribute to transmission clusters, other factors such as prior transmission in home regions or reactivation could not be ruled out in our study population, as it requires more and robust data, including data on precise location of the detention centers, prior history of TB infection among the study population and a clear baseline data on the TB transmission rates in similar populations. While the possibilities of mixed TB transmission cluster are also possible, lack of such comparative data precludes conclusive determination on whether the observed cluster rates were due to reactivation or reinfection. The clustering of isolates might also suggest possible transmission links among individuals; however, due to the cross-sectional nature of this study and the lack of temporal and spatial epidemiological data, we could not determine whether transmission occurred within detention centers or prior to individuals’ arrival there. Moreover, application of ≤12 SNP clustering threshold in our study might highlight its relevance to the observed transmission clusters. Unlike the study by Abyot et al [34], the TB clustering in our study population was relatively higher, possibly attributed to differences in the study population dynamics and mobility, epidemiological contexts and differing exposure histories.

While our study marks a pioneering application of WGS to delve into the drug susceptibility patterns and transmission clusters of *M. tuberculosis* among returnees, it is crucial to approach our results with consideration of specific limitations. Primarily, the study was conducted with a notably limited number of returnees, offering a snapshot rather than a comprehensive representation of the overall challenge. Secondly, due to resource constraints, minimum inhibitory concentration (MIC) testing was not performed for the *M. tuberculosis* isolate containing drug resistance-conferring gene mutations. Thirdly, no female participants were enrolled, as none of the screened female returnees tested positive for TB. Additionally, the intricacies of data collection were compounded by challenges faced by returnees in accurately describing their place of detention during the data collection. Significantly, the study participants primarily comprised individuals unable to reunite with their families and constrained to stay in an isolation center (Chefa Health center) due to internal conflict-afflicted settings in the northern part of Ethiopia during the study period. These contextual factors underscore the necessity for a cautious interpretation, recognizing the unique circumstances that shape the scope and outcomes of this study.

## Conclusion

Overall, this study provides a comprehensive analysis of drug resistance patterns and lineage distribution of *M. tuberculosis* in KSA returnees to Ethiopia, revealing a notable drug resistance mutations and multi-drug resistant isolates through advanced WGS. Lineage 4 of *M. tuberculosis* showed a significantly high prevalence of drug resistance. Despite lack of our ability to assess the living conditions of the study participants in the detention centers or other potential sources of exposure and the lack of temporal and epidemiological data to distinguish recent transmission from reactivation of latent TB infections, the observed evidence of recent transmission cluster is substantiated by genetic and epidemiological link analyses. In light of these findings, we advocate for the implementation of robust government policies and strategies for screening and diagnosing returning migrants. Such proactive approach is critical to controlling drug-resistant TB transmission during the reintegration of returnees into the community, significantly contributing to safeguarding public health and curtailing the spread of TB within the population. Overall, transmission clusters are based on genomic clustering and are suggestive of possible transmission patterns of *M. tuberculosis* rather than definitive transmission. The need for longitudinal studies, incorporating temporal and spatial epidemiological data is warranted to strengthen the interpretation of transmission clusters observed in detention centers.

## Supporting information

S1 File S1_Socioclinical data, S2_DNA_QC, S3_WGS_QC, S4_DR_Mutations, S5_lineages and sub_lineages, S6_SNP distance matrix, S7_clusters and S8_transmission link. (XLSX)

S1 Table Socio-demographic characteristics and clinical presentation of study participants; August-December 2022, Addis Ababa. (DOCX)
