# Supplementary material for: Genotypic drug resistance and transmission clusters of Mycobacterium tuberculosis isolates among Ethiopian returnees from Saudi Arabia
Source: PLoS One. 2025 Apr 16;20(4):e0318743. doi: 10.1371/journal.pone.0318743 (PMC12002542; doi:10.1371/journal.pone.0318743)
Supplement: S1 Table — (DOCX) [file pone.0318743.s002.docx]

**Supplementary Table**

Socio-Demographic Characteristics and Clinical Presentation of Study Participants; August- December 2022, Addis Ababa

| **Variables** | | **Frequency, N (%)** |
| --- | --- | --- |
| **Age Group**  (years) | 18 - 34 | 82 (93.2) |
|  | 35 - 49 | 6 (6.8) |
| **Gender** | Male | 88 (100) |
| **Persistent cough for ≥ 2 weeks** | Yes | 81 (92) |
|  | No | 7 (8) |
| **Low-grade fever** | Yes | 68 (72.3) |
|  | No | 20 (22.7) |
| **Night sweating** | Yes | 69 (78.4) |
|  | No | 19 (21.6) |
| **Loss of Appetite** | Yes | 61 (69.3) |
|  | No | 27 (30.7) |
| **Previous TB treatment history** | Yes | 7 (8) |
|  | No | 81 (92) |
